# Prevalence and patterns of pediatric surgical pathologies in three referral hospitals in Cameroon

**DOI:** 10.3389/fsurg.2025.1566448

**Published:** 2025-05-21

**Authors:** Pascal Nwandum, Pauline Mantho, Leslie Tasha Mbapah, Edna Fongod, Tchiazah Ayaba, Midrelle Syntyche Tsague, Cyril Egbe Obi, Dieudonne Njibili Oponde, Valerie Egbe Mbi, Elvis Tanue, Marcelin Ngowe Ngowe

**Affiliations:** ^1^ Faculty of Health Sciences, University of Buea, Buea, Cameroon; ^2^ Department of Surgery and Specialties, Faculty of Medicine and Pharmaceutical Sciences, University of Douala, Douala, Cameroon; ^3^ Department of Surgery, University of Pittsburgh School of Medicine, Pittsburgh, PA, United States

**Keywords:** prevalence, pediatric, surgery, patterns, surgical pathology, children, Cameroon

## Abstract

**Background:**

Little is known about the spectrum and caseload of surgically managed pediatric conditions in Cameroon. This study describes the prevalence and patterns of pediatric surgical pathologies in three major hospitals across two regions in Cameroon.

**Methods:**

A hospital-based retrospective descriptive analysis of children aged ≤18 admitted for surgical conditions at the General Hospital, Douala, Laquintinie Hospital, Douala, and the Regional Hospital, Buea, from January 2019 to December 2021. Patient files and theatre registers were reviewed. A data extraction form was used to collect socio-demographic and clinical data. Data was analyzed using SPSS version 25.

**Results:**

There were 1,526 pediatric surgical cases, which made up 12.6% of all pediatric admissions during the study period. There was a male predominance of 63.5%. The age group 6–12 years was the most frequent, 26.2%. Neonates represented 7.3% of all pediatric surgical patients. Most of the patients presented as an emergency, 58.0%. About 36.0% of patients presented late with symptoms lasting more than 1 week to several months, with 14% already having complications on admission. Pediatric injuries (39.8%), congenital anomalies (25.6%), and gastrointestinal surgical pathologies (14.8%) were the most observed patterns of presentation.

**Conclusion:**

Surgical pathologies constitute a significant proportion of pediatric admissions. Injuries, congenital anomalies, and gastrointestinal surgical pathologies are most frequently observed. Most children with surgical pathologies present late to the hospital with complications. Tailored measures to mitigate the burden of pediatric surgical pathologies are needed.

## Background

An estimate by the Disease Control Priority Project stated that surgically correctable pathologies constitute 11% of the total burden of disease in low- and middle-income countries (LMICs) (1). It is also estimated that 85% of children in LMICs will have a surgically treatable disease or condition by the age of 15 years (2, 3). More than 95% of global childhood deaths occur in LMICs (4). In these countries, the pediatric population constitutes more than 50% of the population (2). In 2020, the population of Cameroon was about 26.5 million, with the age group 0–19 comprising 54% of the total population (5). Surgical conditions have a critical impact on children's health (6).

Although surgical conditions in adults are increasingly recognized as a significant healthcare challenge in resource-poor countries, the spectrum and burden of surgical conditions among children remain poorly defined, with inadequate data on pediatric surgical conditions (7–9). Research on children's health in Cameroon has so far focused on describing and estimating the burden of non-surgical infectious and metabolic diseases such as malaria, HIV/AIDS, and malnutrition (10); consequently, most government policies and international aid have focused on curbing and improving the outcomes of these diseases (10).

Limited data exists regarding the range and impact of surgical conditions in children (11). This has led to a lack of focus on specialists, infrastructure, and financial support (4, 12, 13), even though management costs are high and facilities and staff are inadequate. To achieve global health coverage, precise data should be made available about the patterns and burden of surgical diseases in the pediatric age group in Cameroon to help prioritize resources and improve treatment outcomes.

There is limited information on the burden and presentation patterns of surgical conditions in children within Sub-Saharan Africa. The few existing country-specific data show significant variations in their patterns and prevalence, which are quite different from those reported in western countries (14, 15). Most studies in Cameroon have described the patterns and burdens of isolated surgical conditions in children. Thus, it is difficult to estimate the total burden of all pediatric surgical pathologies presenting in major hospitals in Cameroon. In a study conducted in 2013 by Chichom-Mefire A. and Marcus Fokou, 16% of all emergency injury cases occurred in patients aged 0–15, with up to 29% of the cases admitted to the surgical ward (4). In another study in Yaoundé, Cameroon, it was reported that children comprised 19% of all trauma cases (16). A cross-sectional study at Laquintinie Hospital Douala found a hospital prevalence of 42.4% for life-threatening pediatric emergencies (17). However, the study did not provide specific data on pediatric surgical emergencies.

Data regarding the overall prevalence, patterns, and burden of surgical conditions in this population are limited. This knowledge gap hinders policymakers, administrators, and international aid organizations from accurately assessing the health burden of surgically correctable pathologies in the pediatric population in Cameroon. The Lancet Commission on Global Surgery and Bickler in the 1990s emphasized the urgent need for data on pediatric surgical needs in low- and middle-income countries (LMICs). This spectrum encompasses a wide range of conditions with varying prevalence across different sociodemographic groups. To the best of our knowledge, there is limited data available for Cameroon. Therefore, it is crucial to gather data on the prevalence and patterns of surgical presentation of pediatric surgical pathologies in Cameroon. This data will assist policymakers and healthcare workers in identifying areas with the greatest need, better allocating resources, and improving the management of pediatric patients.

## Materials and methods

### Study design

We conducted a retrospective study, reviewing files of pediatric surgical patients admitted at the General Hospital Douala, Laquintinie Hospital Douala, and the Buea Regional Hospital over a period of 3 years (from January 1st, 2019, to December 31st, 2021).

### Study area and setting

The General Hospital Douala is a tertiary-level referral university teaching hospital with a capacity of approximately 350 beds. It offers a comprehensive range of medical services, including surgery, pediatrics, obstetrics and gynecology, nephrology and hemodialysis, intensive care, emergency medicine, internal medicine, ENT, ophthalmology, oncology and radiotherapy, medical imaging, laboratory, and pharmacy.

Laquintinie Hospital Douala is a secondary referral healthcare facility with a capacity of about 200 beds. Its services span a wide range of specialties organized within different departments. The surgical department has seven subdivisions, including a fully functional pediatric surgical unit operational since 2019, and is staffed by two full-time pediatric surgeons. Other subdivisions include general surgery, neurosurgery, ENT, ophthalmology, urology, orthopedics, and traumatology. In addition to the two full-time pediatric surgeons, the surgical department consists of four traumatologists, three urologists, three neurosurgeons, and three visceral surgeons. All pediatric and neonatal surgical patients are hospitalized in a separate pediatric surgical ward.

The Buea Regional Hospital (BRH) is a secondary healthcare facility serving as a referral center for 18 health districts. It has a capacity of about 100 beds. It includes facilities such as a radiology unit, internal medicine unit, obstetrics and gynecology unit, pediatric and neonatal unit, HIV treatment center, physiotherapy, general surgical ward, and hemodialysis center. Each unit is staffed by full-time specialists, general practitioners, and nurses. There is one specialist anesthetist and two nurse anesthetists. The general surgical ward has a capacity of 28 beds for both adult and pediatric patients. Currently, there are two general surgeons but no pediatric surgeon.

### Study population and sample

The study population included all children ≤18 years admitted in the selected hospitals within the study period. The sample was pediatric patients with surgical conditions among all pediatric admissions.

### Inclusion criteria

All pediatric patients' records with surgical pathologies admitted at the above-mentioned selected hospital during the study period.

### Exclusion criteria

Records with incomplete socio-demographic and clinical data were excluded.

### Study procedure

After obtaining approval for the study, patient records and folders in surgical, pediatric, and/or neonatal units within the study period were retrieved and reviewed for completeness. Complete records were entered into a data collection sheet. After the data collection, the records and files were returned to the respective archives.

### Data collection and statistical analysis

Data was collected using an online electronic form (Kobotoolbox) and subsequently exported to Microsoft Excel 2010. The Statistical Package for Social Sciences (SPSS) version 25.0 was employed for data analysis. Categorical variables were expressed as proportions and compared using the Chi-square test, while continuous variables were presented as means or medians, as appropriate. A *p*-value of ≤0.05 was considered statistically significant.

## Results

A total of 12,242 files were reviewed, of which 10,539 pediatric non-surgical admissions were excluded, and 1,526 records met our inclusion criteria for surgical pathologies. Of these, 177 records were excluded due to incomplete data, resulting in 1,349 pediatric surgical records being effectively reviewed. The excluded records were <5% of the total records; therefore, the effect of excluding 177 records on the results was negligible.

The prevalence of pediatric surgical conditions was calculated as follows:

$$\begin{array}{c} P=\frac{\mathrm{Number}\;\mathrm{of}\;\mathrm{pediatric}\;\mathrm{surgical}\;\mathrm{admissions}}{\mathrm{Total}\;\mathrm{number}\;\mathrm{of}\;\mathrm{pediatric}\;\mathrm{admissions}}\\ \;\;\;\;=1526/(10539+1526)\;=1526/12065\;=\;12.6\text{\%} \end{array}$$

**Figure 1:**
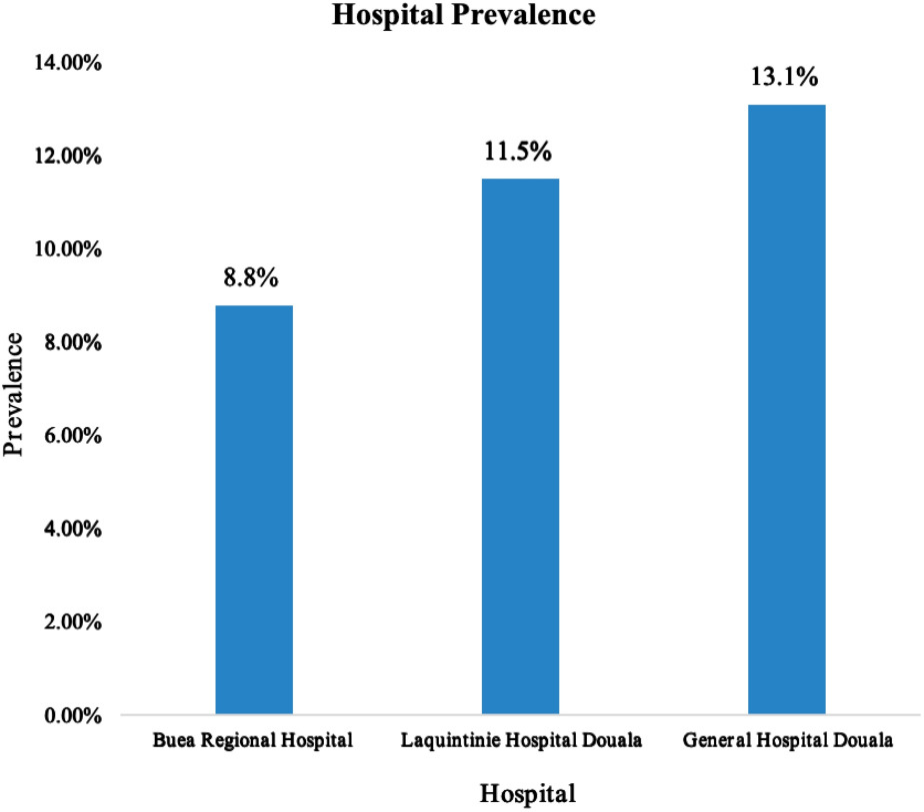
Hospital Prevalence of pediatric surgical conditions.

Thus, the prevalence of pediatric surgical conditions in the three referral hospitals in Cameroon was 12.6% (95% CI: 12.1–13.3). The Douala General Hospital recorded the highest prevalence of pediatric surgical pathologies at 13.1% (95% CI: 12.1–14.1) (Figure 1).

Most pediatric surgical admissions (44.0%) were recorded at the Douala General Hospital, with 74.7% of these patients residing in the Littoral Region of Cameroon. The age group of 6–12 years constituted the largest proportion (26.2%) of patients admitted with surgical conditions, while neonates accounted for 7.3% of the total surgical patients. Males represented 63.5% of cases, resulting in a male-to-female ratio of 1.74:1. A total of 58.0% of pediatric surgical patients were admitted as emergencies. Approximately 43.4% of symptoms and signs on admission lasted less than 24 h from onset to hospital consultation, whereas 35.3% of patients presented with symptoms persisting for more than one week to several months. Just over one-fifth of pediatric surgical patients were admitted as referrals from other hospitals, with 80% originating from primary-level health facilities and 18.5% from secondary-level health facilities in Cameroon. See Table 1.

**Table 1:** Sociodemographic and general characteristics of pediatric surgical patients in three referral hospitals in Cameroon (*N* = 1,349).

Characteristic	Category	Frequency	Percentage (%)
Hospital	LHD	462	34.2
	GHD	593	44.0
	BRH	294	21.8
Region of residence	Southwest	315	23.4
	Littoral	1,008	74.7
	Others	26	1.9
Age group of patient	0–28 days	99	7.3
	>28 days–2 years	246	18.2
	>2 years–6 years	308	22.8
	>6 years–12 years	353	26.2
	>12 years–18 years	343	25.4
Sex	Male	856	63.5
	Female	493	36.5
Mode of admission	Emergency	783	58
	Elective	566	42
Presented as referrals from other hospitals	From primary level hospital	255	81.5
	From secondary level hospital	58	18.5
Duration of symptoms	<24 h	585	43.4
	1–3 days	159	11.8
	4–7 days	128	9.5
	>7 days	477	35.3
Pre-existing diseases	Present	23	1.7
	Absent	1,326	98.3
Number of previous surgical interventions	One	42	3.1
	Two	11	0.8
Method of treatment	Operative	957	72.6
	Conservative	388	29.4
	Intensive care	27	2.0
Mode of treatment	Elective	950	70.4
	Emergency	399	29.6
Time between symptom onset and operative intervention	<24 h	164	17.1
	1–3 days	197	20.7
	4–7 days	120	12.5
	>7 days	476	49.7

LHD, Laquintinie Hospital, Douala; GHD, General Hospital of Douala; BRH, Buea Regional Hospital*.*

**Figure 2:**
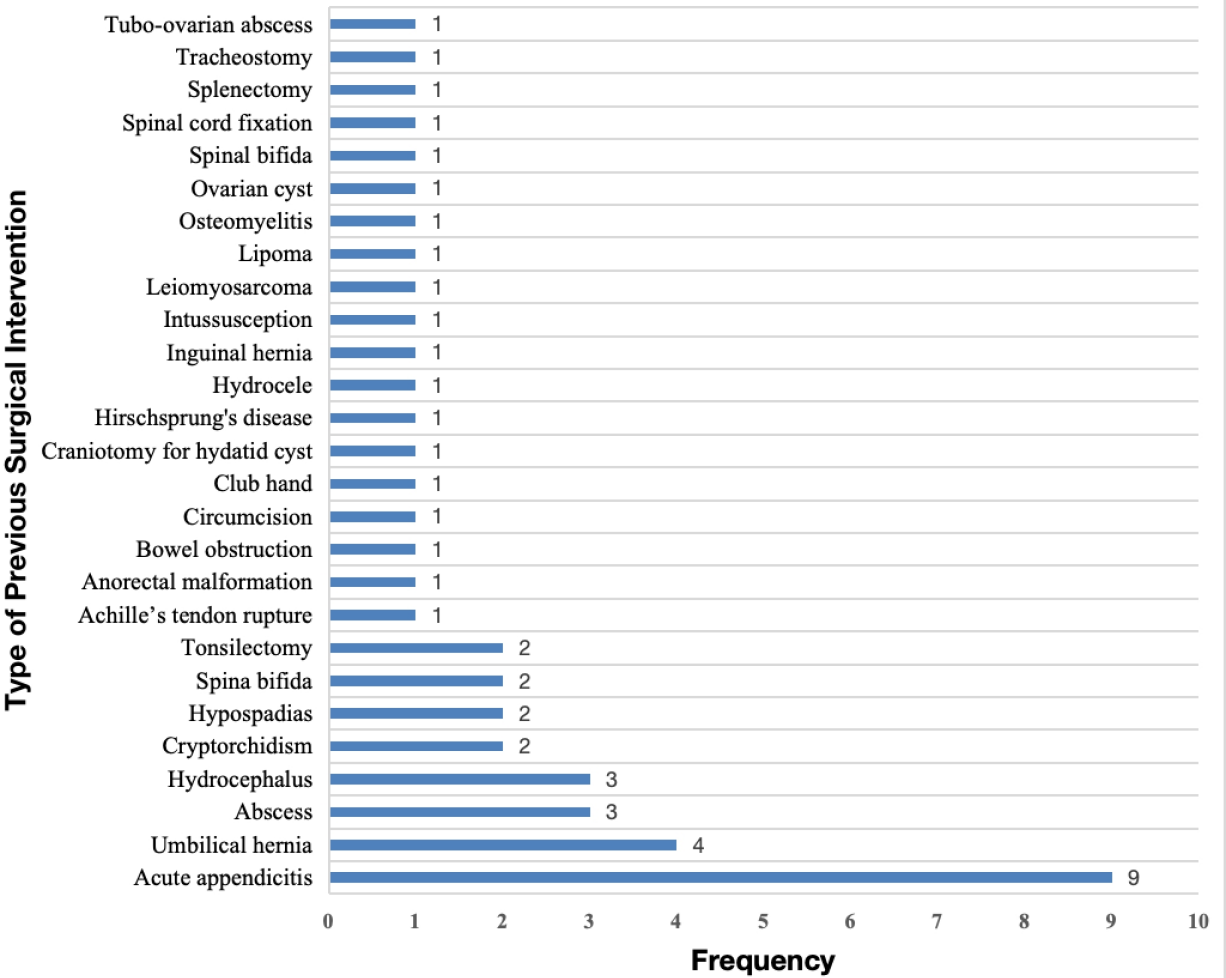
Types of previous surgical interventions in pediatric surgical patients.

Among all pediatric surgical admissions, 1.7% reported a history of preexisting conditions. Congenital malformations were the most frequently reported preexisting medical condition (39.1%), with hydrocephalus being the most common at 66.7%. This was followed by genetic diseases (30.4%), with sickle cell anemia accounting for 100% of these cases, and chronic illnesses (30.4%), including epilepsy (57.1%) and HIV (28.6%). Most patients (94.1%) had no record of previous hospitalization, and 96.1% had no prior surgical intervention (Table 1). Nine patients (19.6%) had previously undergone surgery for acute appendicitis, and four patients (8.7%) for umbilical hernia (Figure 2). Of all admissions, 29.6% were treated as emergencies. The majority (72.6%) of surgical conditions were managed operatively. Half of the operative cases had symptoms lasting more than one week to several months from onset to surgical intervention. About 2.0% of all admitted cases required intensive care during treatment (Table 1).

**Figure 3:**
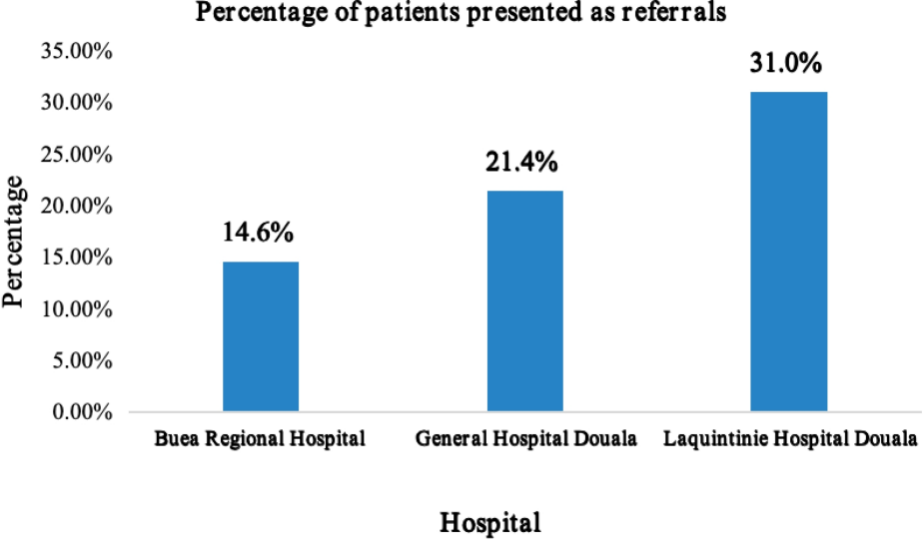
Proportion of patients presenting as referrals from other hospitals.

The Laquintinie Hospital in Douala recorded a statistically significant number of referral cases (31.0%) compared to the other hospitals (*χ*^2^, *p* < 0.001) (Figure 3).

**Figure 4:**
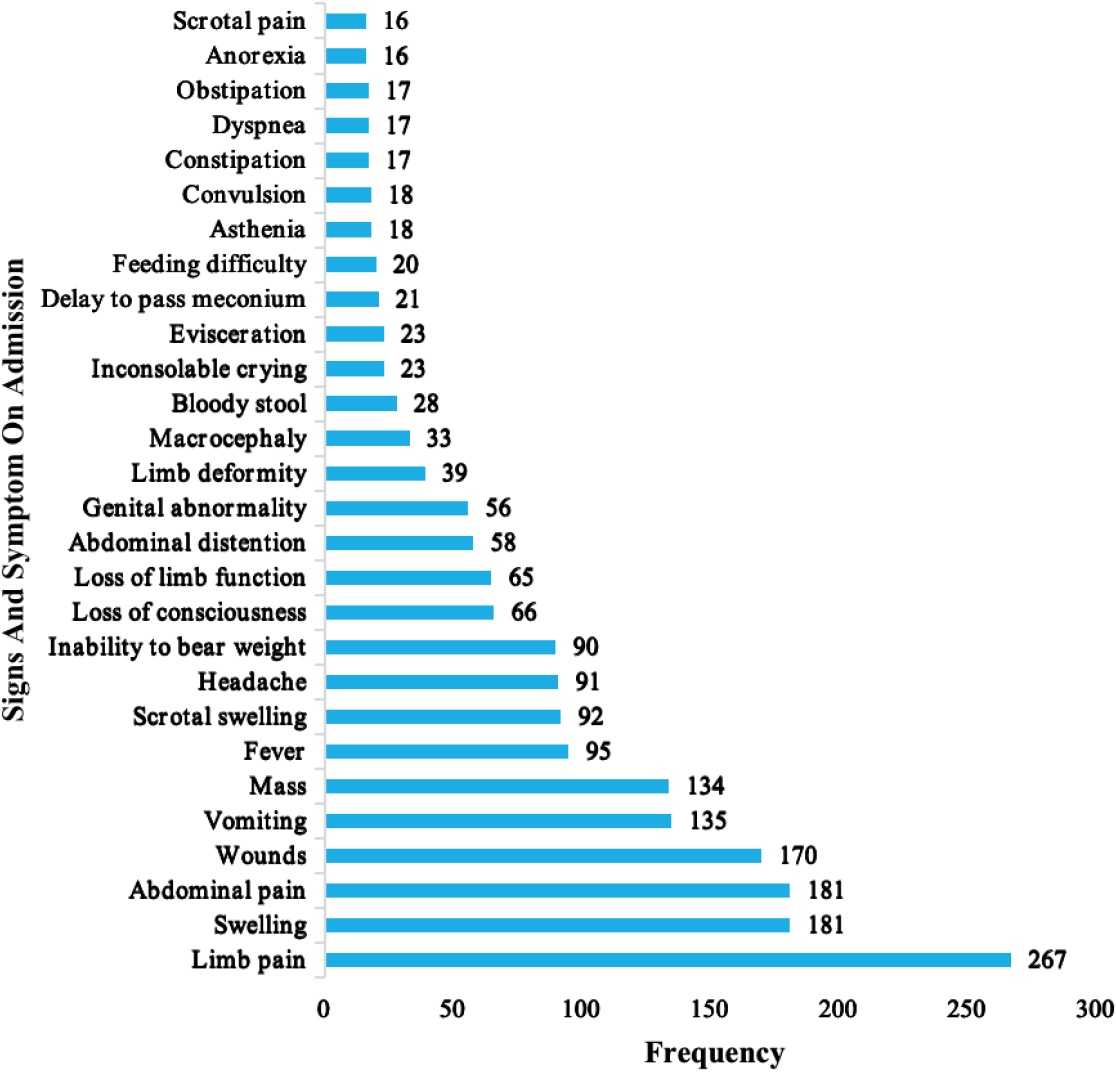
Signs and symptoms of pediatric surgical patients on admission.

The most frequent presenting complaints of the pediatric surgical patients were limb pain 267 (19.8%), swelling 181 (13.4%), abdominal pain 181 (13.4%), wounds 153 (11.3%), and vomiting 134 (9.9%). The common signs and symptoms are presented in Figure 4.

**Figure 5:**
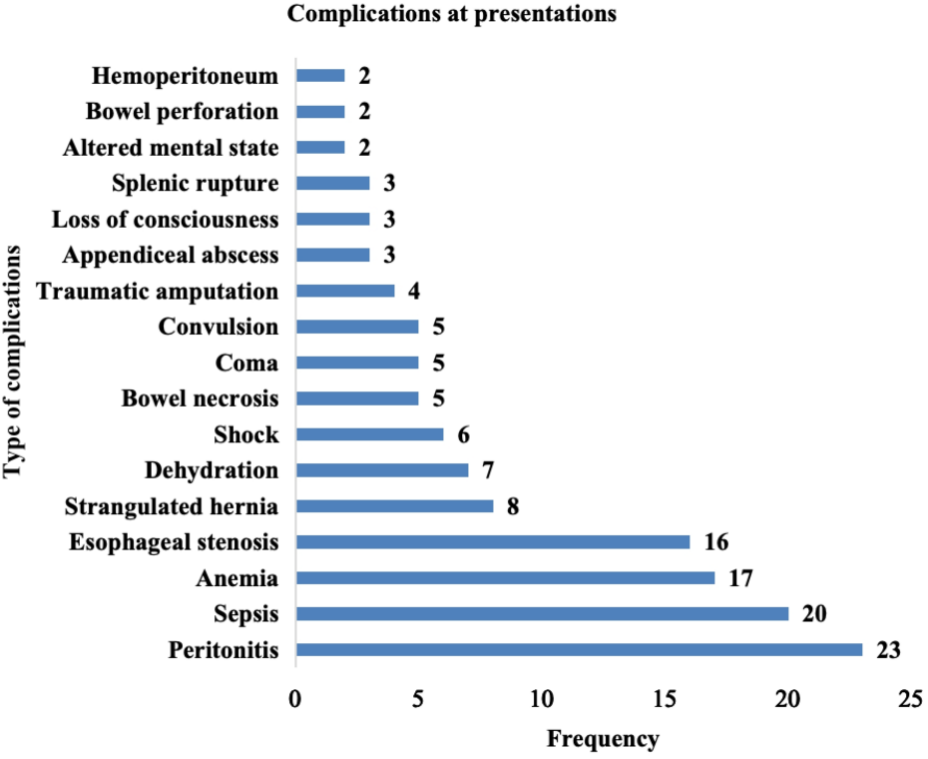
Complications at presentation of pediatric surgical patients.

Complications at presentation were recorded for 180 (13.3%) pediatric surgical patients. Figure 5 shows the complications recorded among pediatric patients presenting for surgical interventions. Peritonitis 23 (12.8%), anemia 17 (9.4%), and strangulated hernia 6 (8.9%) were the most common complications among these children. Complications at presentation correlated with an increased duration of symptoms on admission.

**Figure 6:**
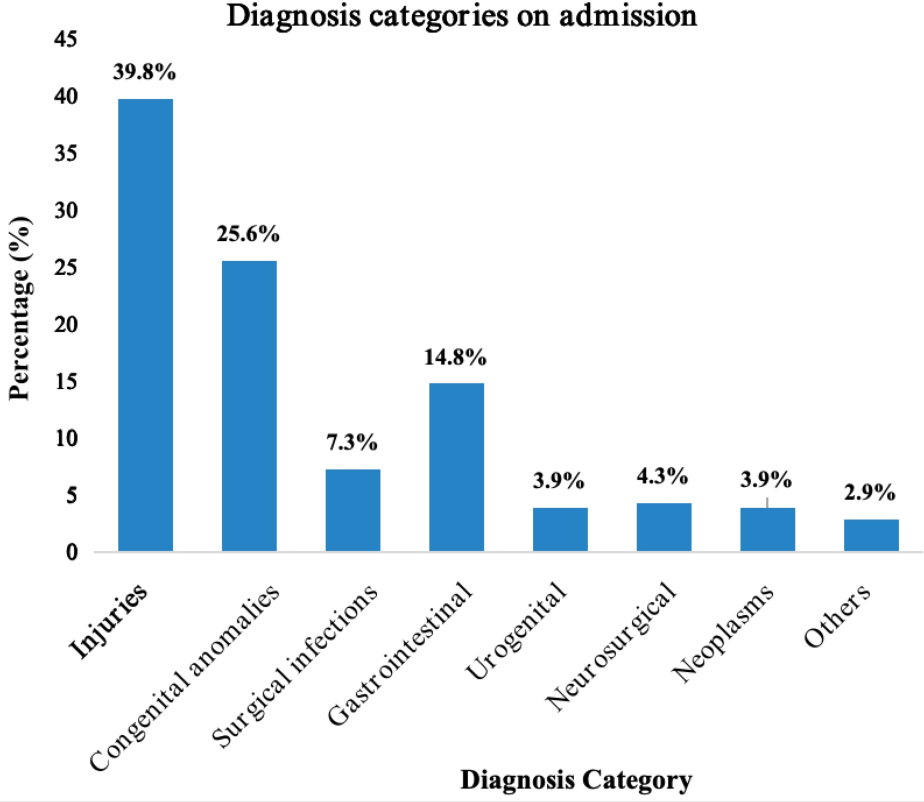
Common diagnosis categories of pediatric surgical patients on admission.

The most common causes of pediatric surgical admissions are illustrated in Figure 6.

Pediatric Injuries (Table 2), 537 (39.8%), were the most common diagnosis of all pediatric surgical conditions observed in the three hospitals. Road traffic accidents, 219 (42.4%), Falls, 161 (31.2%), domestic injuries, 61 (11.8%), and assaults, 32 (6.2%), were the most frequent mechanisms of injuries, resulting mainly in fractures, 246 (46.1%), and head injuries, 184 (34.5%).

**Table 2:** Clinical characteristics of pediatric injuries in three referral hospitals in Cameroon.

Diagnosis	*n* (%)	Most frequent age group	M:F
Burns	47 (8.8)	28 days–2 years	24:23
Fractures	246 (46.1)	6–12 years	149:97
Head injuries	184 (34.5)	12–18 years	110:74
Soft tissue injuries	99 (18.5)	12–18 years	65:34
Dislocations	13 (2.4)	12–18 years	9:4
Blunt abdominal trauma	33 (6.2)	6–12 years	7:4
Penetrating abdominal injuries	3 (0.6)	6–12 years	1:2
Blunt chest trauma	8 (1.5)	6–12 years	3:1
Penetrating chest trauma	6 (1.1)	12–18 years	5:1
Others	17 (3.2)	12–18 years	10:7

Congenital anomalies (Table 3) were the second most observed diagnosis category on admissions after injuries, constituting 25.6% of all surgical admissions. Frequent congenital malformations included inguinal hernia (27%) and hydrocele (18.0%), while hypertrophic pyloric stenosis, omphalocele, gastroschisis, and Hirschsprung's disease accounted for 4.3%, 3.2%, 3.5%, and 2.0%, respectively.

**Table 3:** Types of congenital anomalies in pediatric patients in three referral hospitals in Cameroon.

Diagnosis	*n* (%)	Most frequent age group	M:F
Umbilical hernia	53 (15.4)	6–12 years	29:24
Inguinal hernia	93 (27.0)	2–6 years	79:14
Omphalocele	11 (3.2)	0–28 days	6:5
Gastroschisis	12 (3.5)	0–28 days	6:6
Hirschsprung's disease	15 (4.3)	0–28 days	15:0
Hypertrophic pyloric stenosis	7 (2.0)	28 days–2 years	5:2
Cryptorchidism	42 (12.2)	2–6 years	42:0
Cleft palate/lip	33 (9.6)	28 days–2 years	24:9
Biliary atresia	1 (0.3)	0–28 days	1:0
Duodenal atresia	10 (2.9)	0–28 days	3:7
Hydrocele	62 (18.0)	6–12 years	62:0
Others	37 (10.7)	0–28 days	17:20

Gastrointestinal surgical pathologies (Table 4) constituted 14.8% of all pediatric surgical admissions. The most reported gastrointestinal surgical pathologies were acute appendicitis 75 (37.5%), acute intestinal obstruction 36 (18.0%), and intussusception 31 (15.5%).

**Table 4:** Types of gastrointestinal pathologies in pediatric patients in three referral hospitals in Cameroon.

Diagnosis	*n* (%)	Most frequent age group	M:F
Acute appendicitis	75 (37.5)	12–18years	38:37
Intussusception	31 (15.5)	28 days–2 years	18:13
Acute intestinal obstruction	36 (18.0)	6–12 years	11:7
Toxic substance ingestion	24 (12.0)	2–6 years	19:5
Acute abdomen	23 (11.5)	6–12 years	16:7
Esophageal foreign body	11 (5.5)	2–6 years	9:2
Others	12 (6.0)	12–18 years	5:7

Surgical infections (Table 5) were the fourth reason for pediatric admissions, constituting 7.3% of all admissions. Abscess (30.6%) and osteomyelitis (34.7%) were common types of surgical infections.

**Table 5:** Surgical infection in pediatric patients in three referral hospitals in Cameroon.

Diagnosis	*n* (%)	Most frequent age group	M:F
Abscess	30 (30.6)	12–18 years	13:17
Osteomyelitis	34 (34.7)	12–18 years	19:15
Septic arthritis	12 (12.2)	2–6 years	2:1
Pyomyositis	12 (12.2)	12–18 years	7:5
Cellulitis	16 (16.3)	12–18 years	5:3
Others	12 (0.9)	12–18 years	1:3

Pediatric neurosurgical, urogenital, and neoplasms (Table 6), respectively constituting 4.3%, 3.9%, and 3.9%, were less frequent causes of pediatric admission into the surgical ward.

**Table 6:** Neurosurgical, urogenital, and pediatric neoplasms in pediatric patients in three referral hospitals in Cameroon.

Diagnosis	*n* (%)	Most frequent age group	M:F
Neurosurgical			
Hydrocephalus	36 (62.1)	28 days–2 years	17:19
Spina bifida	19 (32.8)	0–28 days	13:6
Encephalocele	4 (6.9)	0–28 days	0:4
Others	1 (1.7)	2–6 years	0:1
Urogenital			
Hypospadias	16 (30.8)	2–6 years	16:0
Testicular torsion	8 (15.4)	6–12 years	8:0
Anal prolapse	4 (7.7)	2–6 years	3:1
Acute urinary retention	5 (9.6)	2–6 years	4:1
Circumcision's injury	6 (11.5)	2–6 years	6:0
Others	15 (28.8)	2–6 years	11:4
Neoplasms			
Malignant	7 (14.6)	12–18 years	4:3
Benign	41 (85.4)	12–18 years	19:22

We observed a multidisciplinary approach to treating pediatric surgical cases involving various specialties such as general surgery, pediatric surgery, neurosurgery, otorhinolaryngology, and orthopedic surgery. Most cases (465, 34.8%) were managed by general surgeons. Pediatric surgeons were involved in 412 cases (30.9%), neurosurgeons in 208 cases (15.6%), and orthopedic surgeons in 174 cases (13.0%). Anesthesia was administered by a specialist anesthetist in most cases requiring anesthesia, 742 (77.5%).

## Discussion

This study aimed to ascertain the prevalence and patterns of pediatric surgical conditions in three referral hospitals in Cameroon. According to global disease burden data, surgical conditions in children account for 6%–12% of all pediatric admissions in sub-Saharan Africa (18). However, in this study, the proportion of pediatric (0–18 years) surgical admissions among total pediatric admissions was 12.6%, which is slightly higher than the global disease burden estimates. Our findings are consistent with a cross-sectional study in Somaliland, which reported a prevalence of 12.2% (9). Similarly, Stephen W. Bickler et al. reported a prevalence of 11.3% in their study on the epidemiology of pediatric surgical admissions to a referral hospital in The Gambia (8). A study conducted in Nigeria reported a lower prevalence of 8.1% (19).

There was a male predominance with a ratio of 1.74:1. This ratio differs slightly from that reported by E. Abahuje et al., who found a ratio of 2.2:1 in a one-year review of the epidemiology of pediatric surgery in Rwanda (7). Our result is closer to the ratio of 1.4:1 reported by Minakshi Bhosale 2019 in India (20). The male predominance in our study can be attributed to the fact that most frequent admissions were due to pediatric injuries, and males are generally more active, adventurous, and involved in risky activities. The most frequent age group of pediatric patients in the surgical service was between 7 and 12 years (26.2%). Temesgen et al. in Ethiopia (21) reported that the most frequent age group was 3–5 years (72.2%). In our study, the age group 3–5 years (22.8%) was the third most frequent, following the 12–18 years group (25.4%). Neonates constituted 7.3% of all pediatric surgical admissions, making them the least representative age group. This pattern is similar to most studies in LMICs (12, 19, 21).

The most frequent symptoms in this study were limb pain (19.8%), swellings (13.4%), abdominal pain (13.4%), wounds (11.3%), and vomiting (9.9%). These symptom patterns can be explained by the fact that most pediatric surgical admissions were due to traumatic injuries, primarily affecting the limbs. In the Hani Baragwanath experience in South Africa, 61.7% of pediatric surgical patients presented on an elective basis, while 38.3% presented as emergencies (12). In our study, 783 (58.0%) of patients presented as emergencies, with most 477 (35.3%) of their symptoms lasting more than one week to several months from onset to consultation. Additionally, 13.3% of patients had at least one complication at presentation. A total of 313 (23.2%) of all admissions were referrals from other hospitals, with the majority coming from primary-level hospitals and up to one-fifth referred from secondary-level hospitals. Laquintinie Hospital Douala (LHD) registered the highest number of patients referred among the three hospitals. This can be attributed to the fact that LHD has a separate functioning pediatric surgical service with pediatric surgeons and, due to its location in the center of an interurban town, it receives patients from across the nation. The other two hospitals in this study did not have a separate pediatric surgical service; general surgeons conducted pediatric surgical procedures.

Pediatric injuries 537 (38.9%), congenital malformations 345 (25.0%), and gastrointestinal surgical pathologies 200 (14.5%) were the top three diagnosis categories in this study. This pattern differed from that reported by Bhosale et al. in India, where congenital malformation at 90.25% was the most frequent pediatric surgical pathology, followed by surgical infections at 6.2%, and Trauma at 3.4% was the least frequently observed (20). The higher prevalence of pediatric injuries in our study is because trauma cases were admitted directly in the surgical departments of our hospitals, unlike in their setting, where most trauma cases are first admitted at the general surgical ward, with only very complicated cases transferred to pediatric surgical service for better management. Bickler et al., in a study of the epidemiology of pediatric surgical admissions to a government referral hospital in Gambia, reported the pattern: Injuries (46.9%), Congenital anomalies (24.3%), and surgical infections (14.5%) (8). This pattern was quite similar to ours, apart from the fact that we had gastrointestinal surgical pathologies instead of surgical infections as the third most common diagnosis category. Our patterns were, however, precisely the same as that reported in a Rwandan study by E. Abahuje et al. in a one-year review on the epidemiology of pediatric surgery in Rwanda, where they reported Trauma and burns (36.58%), Congenital malformation (23.39%) and Gastrointestinal surgical pathologies (14.76%) (7). From the above observations, we can conclude that the spectrum of pediatric surgical diseases and presentation patterns is similar in many African countries despite their slight differences.

Generally, as reported by the CDC, pediatric injuries are most commonly caused by motor vehicle crashes, suffocations, drowning, poisoning, burns, falls, and assaults (22). In this study, most of the injuries resulted from Roadtraffic accidents 219 (42.4%), falls from height 161 (31.2%), domestic injuries 61 (11.8%), and assaults 32 (6.2%). This was similar to the findings of Nwanna-Nzewunwa et al, in Douala, Cameoon, with most (54%) of children with Road traffic accidents mostly affecting the limbs and pelvis (16). Our study also noticed that injuries occurred mostly in the 12- to 18-year-old. The most observed injuries were fractures (246, 46.1%), head injuries (184, 34.5%), and soft tissue injuries (99, 18.5%). Pediatric burns constituted 47(8.8%) of all injuries. This was similar to the studies by Oyemaechi et al., in 2020 in Malawi, who reported bruises/lacerations/abrasions (27.6%) and fractures (27.1%) (23), and Ali et al., 2024 who reported limb fractures (34.1%) and traumatic brain injury (20.9%) (24) as the most common injuries. Mechanism of burn was difficult to assess in our study since most files lacked this data. A study conducted at the University of Port Harcourt Teaching Hospital revealed that congenital malformations accounted for 88.9% of all pediatric surgical admissions (25).

Neonatal intestinal obstruction (29.4%) and anterior abdominal wall defect (14.2%) were the most frequently reported malformations (25). In another study by Ajanja Samson at the Kenyatta National Hospital, it was noticed that anorectal malformations (19.2%), Anterior abdominal wall defect (17.2%), congenital diaphragmatic hernia (14.4%) and neural tube defects (9.6%) were the most common malformations (26). In this study, the majority of congenital malformations were inguinal hernia 93 (27%), hydrocele 62 (18%), umbilical hernia, and cryptorchidism 42 (12.2%). Gastrointestinal malformations were also seldomly recorded in our study; Omphalocele 11 (3.2%), Gastroschisis 12 (3.5%), Hirschsprung's disease 15 (4.3%), HPS 7 (2.0%) and duodenal atresia 10 (2.9%). Our results differed from those of both studies conducted by Ajanja in Kenya and the Port Harcourt Teaching Hospital in Nigeria. Gastrointestinal surgical pathologies, as reported by E. Abahuje in a study in Rwanda, were the most common pathologies were; intussusception (2.7%), small bowel perforation (2.6%), and appendicular perforation (2.1%) (7). This differed from our study, where we registered that the age group of 12–18 years had the most gastrointestinal surgical pathologies.

The most frequent conditions were acute appendicitis 75 (37.5%), intestinal obstruction 36 (18.0%), intussusception 31 (15.5%), and acute abdomen 23 (11.5%). Urogenital 52 (3.8%), neurosurgical 58 (4.2%), surgical infections 98 (7.1%), and pediatric neoplasms 52 (2.82%) were the least frequently reported diagnostic categories in this study. Within the neurosurgical category, hydrocephalus 36 (62.1%), spina bifida 19 (32.8%), and encephalocele 04 (6.9%) were the most reported conditions. This is similar to a study in southern Nigeria on the burden of pediatric neurosurgical diseases in a rural developing country, which observed proportions of hydrocephalus (9.3%), myelomeningocele (1.8%), and encephalocele (0.9%) as the most frequent causes of pediatric neurosurgical consultations (19).

In this study, abscesses 30 (30.6%) and osteomyelitis 34 (34.7%) were the most frequent infections requiring surgical admissions. Our results were quite similar to those reported by Mariana Gortan et al. in Burundi, where abscesses (29.9%) and osteomyelitis (9.76%) were among the most frequent causes of pediatric visits to the surgical ward (27).

In this study, we observed hypospadias 16 (30.8%), testicular torsion 8 (14.8%), circumcision injuries 6 (11.5%), and acute urinary retention 5 (9.6%) as the most common pediatric urogenital pathologies managed surgically. In contrast, a study on pediatric urological emergencies at the Enugu State Teaching Hospital, Nigeria, found that acute hydrocele (39.0%), urinary retention (24.1%), testicular torsion (13.1%), and circumcision injuries (13.1%) were the most reported pediatric urogenital surgical pathologies (28).

Children have special requirements regarding physiology, anesthesia, and anatomy (29). Laquintinie Hospital Douala was the only hospital in our study with a specialized and fully functional pediatric surgical service staffed by two pediatric surgeons. The other two hospitals lacked pediatric surgeons and a dedicated pediatric surgical service. This explains why the Laquintinie hospital recorded the highest percentage of referred patients compared to the other hospitals. A pediatric surgeon was available in only 30% of the cases in our study. We also observed a multidisciplinary approach in the management of pediatric surgical patients. The majority (72.6%) of our cases were managed operatively.

### Limitations

This study was a hospital-based review of patient records; we relied solely on the recorded data. Long-term complications, disability, and outcomes following discharge could not be assessed.

## Conclusions

Pediatric surgical pathologies constituted a significant proportion of pediatric admissions in three major hospitals in Cameroon, constituting 2 out of every 10 pediatric consultations. Injuries, congenital anomalies, and gastrointestinal surgical pathologies are most commonly observed. Most children present late at definitive treatment centers, the majority with complications. Late presentation are due mostly to; financial contraints, religious and cultural beliefs, scarce and distant pediatric surgical treatment centers and specialists.

## Recommendations

We recommend prompt identification and referral of surgical pathologies in neonates and children by clinicians. Stakeholders should encourage parent awareness initiatives of the need and importance of early consultation of a child when sick.

## Data Availability

The raw data supporting the conclusions of this article will be made available by the authors, without undue reservation.
